# [Retracted] Long non-coding RNA phosphatase and tensin homolog pseudogene 1 suppresses osteosarcoma cell growth via the phosphoinositide 3-kinase/protein kinase B signaling pathway

**DOI:** 10.3892/etm.2025.13002

**Published:** 2025-10-27

**Authors:** Bin Yan, Aikepaer Wubuli, Yidong Liu, Xin Wang

Exp Ther Med 15:4829–4837, 2018; DOI: 10.3892/etm.2018.6021

Following the publication of this paper, it was drawn to the Editor’s attention by a concerned reader that certain of the western blot data shown in Figs. 3C and 4A on p. 4834 and 4835 respectively were strikingly similar to data appearing in different form in an article written by different authors at different research institutes that had already been published in the journal *Oncotarget* prior to the submission of this paper to *Experimental and Therapeutic Medicine*. Upon performing a further independent analysis of the data in this paper, it came to light that certain of the cell migration and invasion assay data in Fig. 2C and D, the flow cytometric data in Fig. 3C and the majority of the cell migration and invasion assay data in Fig. 4C and D had also been published previously in a number of articles that were written by different authors at different research institutes.

In view of the fact that the abovementioned data had already apparently been published previously, the Editor of *Experimental and Therapeutic Medicine* has decided that this paper should be retracted from the Journal. The authors were asked for an explanation to account for these concerns, but the Editorial Office did not receive a reply. The Editor apologizes to the readership for any inconvenience caused.

